# An Artifact-Resistant Feature SKNAER for Quantifying the Burst of Skin Sympathetic Nerve Activity Signal

**DOI:** 10.3390/bios12050355

**Published:** 2022-05-20

**Authors:** Yantao Xing, Yike Zhang, Zhijun Xiao, Chenxi Yang, Jiayi Li, Chang Cui, Jing Wang, Hongwu Chen, Jianqing Li, Chengyu Liu

**Affiliations:** 1 School of Instrument Science and Engineering, Southeast University, Nanjing 210096, China; 230198304@seu.edu.cn (Y.X.); zhijunxiao@seu.edu.cn (Z.X.); chenxiyang@seu.edu.cn (C.Y.); lijiayi@seu.edu.cn (J.L.); 2 Division of Cardiology, The First Affiliated Hospital of Nanjing Medical University, Nanjing 210096, China; zhangyike@njmu.edu.cn (Y.Z.); cuichang@njmu.edu.cn (C.C.); chenhongwu@njmu.edu.cn (H.C.); 3 Division of Nephrology, The First Affiliated Hospital of Nanjing Medical University, Nanjing 210096, China; wj_flagon@163.com

**Keywords:** sympathetic activity (SNA), skin sympathetic nerve activity (SKNA), electrodes, electrocardiogram (ECG)

## Abstract

Evaluation of sympathetic nerve activity (SNA) using skin sympathetic nerve activity (SKNA) signal has attracted interest in recent studies. However, signal noises may obstruct the accurate location for the burst of SKNA, leading to the quantification error of the signal. In this study, we use the Teager–Kaiser energy (TKE) operator to preprocess the SKNA signal, and then candidates of burst areas were segmented by an envelope-based method. Since the burst of SKNA can also be discriminated by the high-frequency component in QRS complexes of electrocardiogram (ECG), a strategy was designed to reject their influence. Finally, a feature of the SKNA energy ratio (SKNAER) was proposed for quantifying the SKNA. The method was verified by both sympathetic nerve stimulation and hemodialysis experiments compared with traditional heart rate variability (HRV) and a recently developed integral skin sympathetic nerve activity (iSKNA) method. The results showed that SKNAER correlated well with HRV features (r = 0.60 with the standard deviation of NN intervals, 0.67 with low frequency/high frequency, 0.47 with very low frequency) and the average of iSKNA (r = 0.67). SKNAER improved the detection accuracy for the burst of SKNA, with 98.2% for detection rate and 91.9% for precision, inducing increases of 3.7% and 29.1% compared with iSKNA (detection rate: 94.5% (*p* < 0.01), precision: 62.8% (*p* < 0.001)). The results from the hemodialysis experiment showed that SKNAER had more significant differences than aSKNA in the long-term SNA evaluation (*p* < 0.001 vs. *p* = 0.07 in the fourth period, *p* < 0.01 vs. *p* = 0.11 in the sixth period). The newly developed feature may play an important role in continuously monitoring SNA and keeping potential for further clinical tests.

## 1. Introduction

Cardiovascular diseases (CVDs) are the biggest killer of people globally, accounting for 32.1% of the death cases [1]. With the aggravation of aging [2], the prevention and treatment of CVDs have become a global problem. Some CVDs are manifested as symptomatic cardiac autonomic function neuropathy [3], with sympathetic and vagal innervation disorder or structural damage. Therefore, the evaluation of sympathetic function is of great significance [4]. Microneurography is the gold standard for estimating sympathetic nerve activity (SNA) [5], but it is invasive and rarely used in the clinical scene [6]. Heart rate variability (HRV) is a non-invasive method of assessing SNA [7]. HRV requires proper sinus node function [8], and it is not practicable for patients with atrial fibrillation or premature beat because their rhythm is not sinus rhythm. In addition, HRV cannot reflect the dynamic changes of SNA because of their indirect parameters from segments that last 5 min. Therefore, these methods have some limitations for daily human monitoring applications. The research on electrodermal activity (EDA) provides another possibility for non-invasive SNA evaluation [9], but EDA affected by sweat may lead to some individualized differences in evaluation features [10]. As a non-invasive and real-time method for assessing SNA, skin sympathetic nerve activity (SKNA) has been applied in many clinical events [11,12] and has great potential. It has become a research hotspot in SNA evaluation.

**Figure 1:**
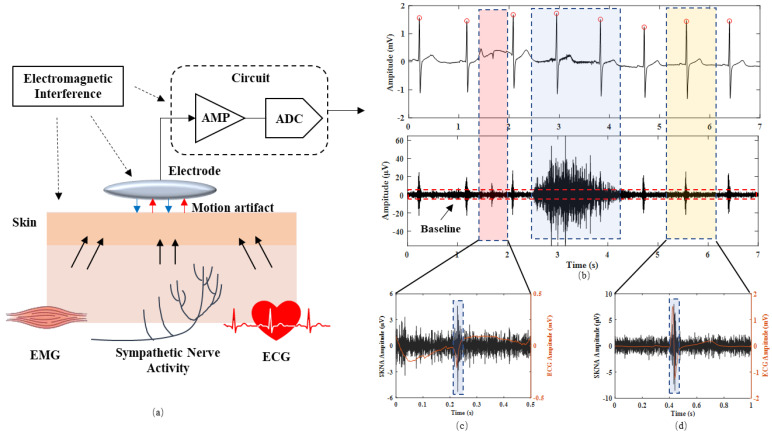
(**a**) The chain of SKNA signal transmission and acquisition. (**b**) Representative examples of acquired signals: the above figure shows the raw signal and the following figure shows SKNA after filtering. (**c**) The step signal artifact. (**d**) The ECG artifact.

As shown in Figure 1, SKNA is obtained by collecting biopotential signals on the body surface using electrode sensors at high sampling frequency (>2000 Hz) [13]. This method does not require additional inducing conditions and is easy to implement. The amplitude range of SKNA signals is much lower than those of electrocardiogram (ECG), ranging from 0.5 to 80 µV, according to the experiments from our previous work [13] as well as from [14,15]. The sympathetic nerve arises from the spinal cord. In particular, the stellate ganglion sends post-ganglionic sympathetic fibers to the heart and skin. The electrodes measure the subcutaneous sympathetic nerve activity to reflect the cardiac sympathetic nerve activity. Therefore, SKNA is inevitably disturbed by other noises, such as ECG and electromyogram (EMG), resulting in various artifacts in the signal [16]. The electrode-skin interface impedance and skin surface conductivity also affect the signal quality [17]. In addition, power line interference and amplifier saturation distortion have a negative impact on SKNA signal acquisition [18]. Therefore, SKNA has some special characteristics: low amplitude, high noise, and high randomness due to the above factors.

There are still some challenges to be addressed in this approach because of the characteristics of SKNA. First, SKNA is aperiodic and has high randomness because it changes with SNA. Therefore, detecting nerve bursts is beneficial to understanding the changes in SKNA. At this point, the nerve bursts are often discriminated with empirical thresholds [19,20]. With the method based on thresholds, it is easy to cause several misjudgments due to the low signal-to-noise ratio of SKNA. The second challenge comes from the quantization of SKNA. Some researchers assess SNA by calculating the average energy of SKNA [21,22]. Because of noise, signal extraction is easily affected, especially in providing sympathetic-related information. Therefore, it is necessary to further process the obtained signals in order to extract key information from SKNA effectively.

This study aims to develop an artifact-resistant feature to quantify the SKNA signal. We expect this feature to show good performance in short-term and long-term SNA evaluation. The main contributions of this paper can be summarized as follows:

(1) To more accurately detect the burst area, we proposed a method based on TKE operator and envelope and integral signal to detect the burst area. In addition, we proposed to discriminate the ECG artifacts based on QRS complexes.
(2) To accurately quantify the signal, we proposed a new feature, SKNAER, for SNA evaluation based on the detected burst area. We compared SKNAER with aSKNA in the hemodialysis clinical experiments. HRV features related to SNA were calculated simultaneously based on ECG for the comprehensive comparison.

## 2. Materials and Methods

### 2.1. Experimental Design

#### 2.1.1. Experimental Setup

**Figure 2:**
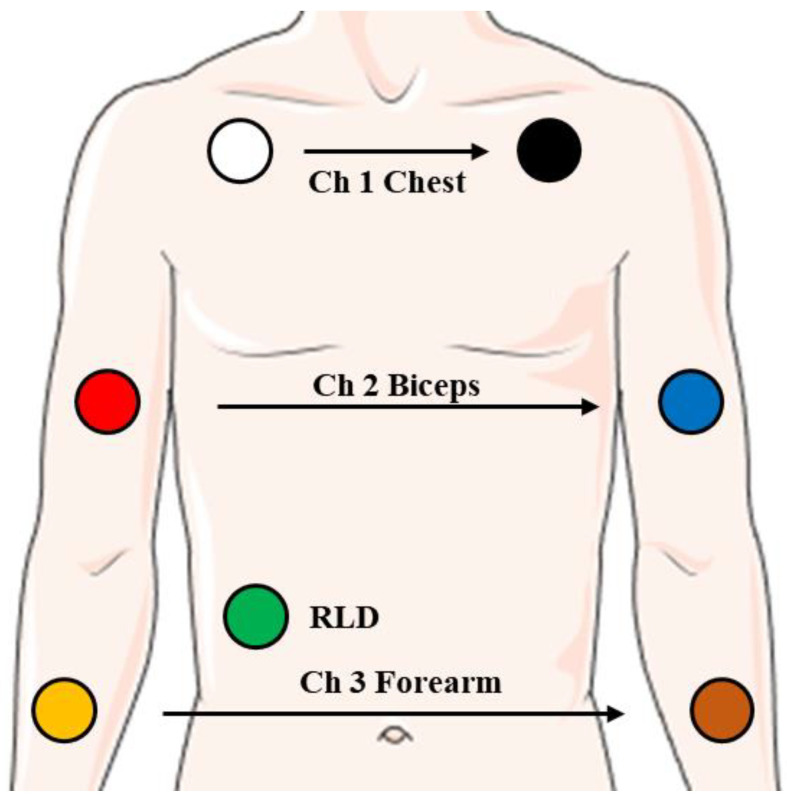
The electrode placement position in the experiment. In Experiment 2, the signal is only collected from channel 1.

PowerLab Data Acquisition Hardware Device (ADInstruments, Lexington Drive Bella Vista New South Wales, Australia) is the signal acquisition system used in the experimental protocol. The data acquired from the signal acquisition system were analyzed by LabChart pro 8 software (ADInstruments, Australia). All the experimental results were imported into MATLAB^®^ (R2019) for further processing and illustrating. The sampling frequency was set to 8 kHz. Two types of experiments were conducted to verify the reliability and effectiveness of the method, including experiments in laboratory environments and clinical experiments. In this work, the signal measurements in the laboratory were carried out in a noise-free sound insulation room, with a temperature of 25 °C and humidity of 50%. The clinical experiments were carried out in the operation room, with a temperature of 25 ℃ and humidity of 50%. Seven surface electrodes (3M^®^) were placed on the chest, biceps, forearm, and right abdomen. The positions of electrodes on the body are shown in Figure 2. The sensor positions of the two experiments were the same. Considering the distribution of muscle tissue on the body surface, we assume that ch1 is high signal quality, ch2 is medium signal quality, and ch3 is low signal quality.

#### 2.1.2. Experimental Protocol

Table 1 summarizes the demographic information of the experiments. Ten healthy subjects without CVDs in Experiment 1 and twenty clinical subjects in Experiment 2 from the First Affiliated Hospital of Nanjing Medical University participated in this study. The healthy subjects’ average age, height, and weight without CVDs were 25.1 ± 4.6 years old, 173.2 ± 6.5 cm, and 71.0 ± 13.6 kg. The clinical subjects’ average age, height, and weight were 58.9 ± 14.6 years old, 170.2 ± 10.3 cm, and 70.5 ± 13.9 kg. Written and informed consent was obtained from each patient. The Ethics Committee has approved the patient experimental protocols of the First Affiliated Hospital of Nanjing Medical University, understudy number 2020-SRFA-183.

**Experiment 1: Standard SKNA signal**

We enrolled ten healthy volunteers for signal recording during the cold-water pressor test (CPT) [23] and the Valsalva maneuver (VM) [24]. The two experiments consisted of three steps. The subjects were required to stay in a seated position for a half minute during the first step. This step aimed to eliminate the interference of signal recording at the beginning of the equipment and record the baseline waveform for each subject. The second step was the VM and CPT, the standard procedures for triggering sympathetic discharges. In VM, the subjects were directed to close the glottis after deep inspiration for 30 s. Then, the subjects were monitored for an additional 30 s after directed exhalation. The subjects were guided to use abdominal breathing and avoid unnecessary chest movements. The CPT was performed by placing the subject’s left hand up to the wrist in iced water for one minute. The hand was taken out of the iced water after a minute. These experiments were non-invasive and non-drug experiments to change the autonomic nervous system activity. Each subject was required to repeat each task 10 times. A two-minute control and recovery period was recorded for both maneuvers.

**Experiment 2: Clinical SKNA signals**

The clinical signals were recorded in an operating room. The data of uremic patients during hemodialysis were recorded. Changes in blood volume during hemodialysis can affect the autonomic nervous system. Firstly, the subjects stayed in the supine position before the operation and started recording data simultaneously. After the nurse punctured the arteriovenous fistula of the patients, the patients’ blood was drawn out of the body. Then, the blood was exchanged in the dialyzer to remove the toxin. Finally, the processed blood was fed into the patients’ bodies. The whole hemodialysis process lasted approximately 4 h. It is worth mentioning that the subjects were patients who needed hemodialysis for their chronic kidney disease. The experiment was an observational data recording experiment and had no additional impact on patients. Data recordings were stopped after hemodialysis. The clinical signal only collected the data from channel 1. On the one hand, the interference of data acquisition on clinical patients should be avoided as much as possible. On the other hand, the emphasis of Experiment 2 is different from that of Experiment 1, and more emphasis is placed on verifying the clinical application effect. The patients were asked to stay supine and avoid unnecessary movement during the recording. Electronic instrument usage, which could produce signal artifacts, was avoided during recordings.

### 2.2. Burst Detection Method with iSKNA

The burst can be defined as a period of continuous biopotential signal with amplitude higher than the baseline. Therefore, the signal state can be expressed using the following formula:

(1) $$s\sim \{\begin{array}{l} 1,\;\;\;if\;h\left(x\right)\geq \tau \;\\ 0,\;\;\;if\;h\left(x\right)<\tau \end{array}$$

where *s* is the state of the signal and *h*(*x*) is the statistical feature of the signal. When the statistical characteristic is greater than the threshold *τ*, it is considered that the signal state at this time is burst. Otherwise, it is the baseline.

**Figure 3:**
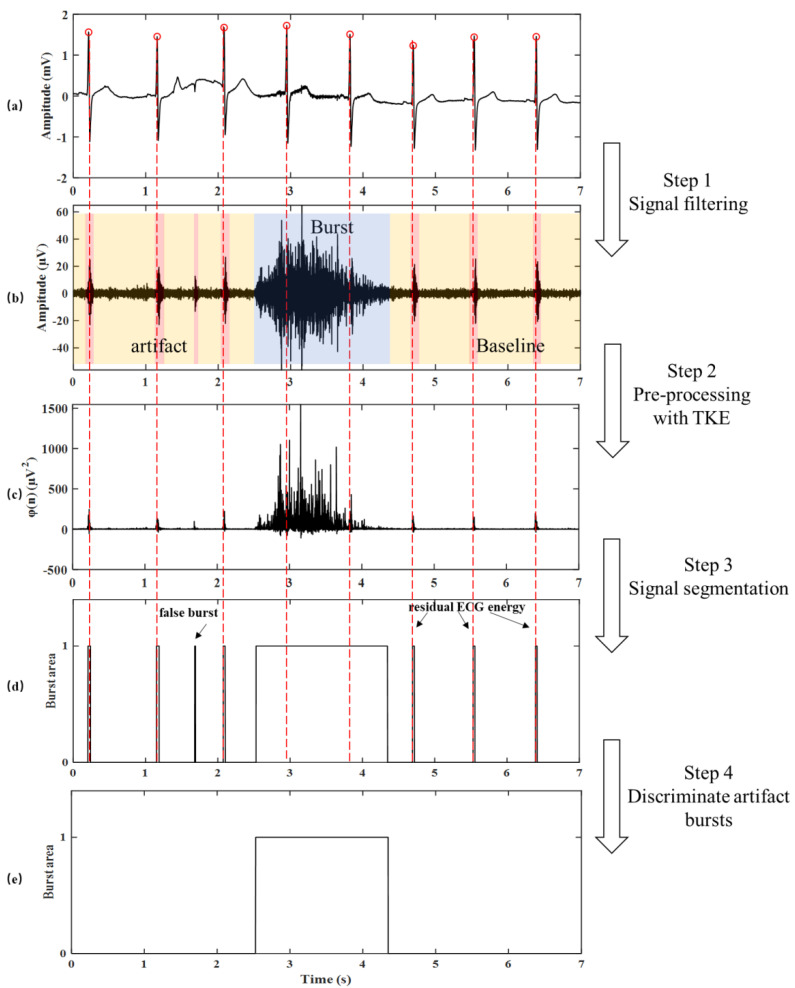
Representative examples of signal preprocessing and segmentation processes using the QRS information complexes and a sensitive threshold. (**a**) The raw signal. (**b**) The filtered SKNA signal. (**c**) The preprocessed signal after TKE operator. (**d**) The segmented burst area based on envelope and integral signal. (**e**) The final segmented burst area. The burst was in the blue box, the baseline was in the yellow box, and the artifact was in the red box.

As shown in Figure 3a,b, the measured signal was first band-pass filtered (from 500 to 1000 Hz). Then, rectified signal was obtained by full-wave rectification [12].

The envelope of the rectified signal was created with a first-order resistance-capacitance integrating the network with a time constant of 0.1 s. After this step, integral skin sympathetic nerve activity (iSKNA) was obtained. At this point, the baseline and burst are often discriminated with a threshold. The threshold is calculated using the following formula:

(2) Threshold=μ+ρ×σ

where *μ* is the mean of iSKNA, *σ* is the standard deviation of iSKNA, and *ρ* is an empirical parameter. Commonly, *ρ* is set to 3 in previous studies [11,12]. One must decrease *ρ* to increase sensitivity for SKNA burst. One must increase *ρ* for higher specificity.

### 2.3. Optimized Burst Detection Method

#### 2.3.1. Teager–Kaiser Energy Operator

Before burst localization, the TKE operator [25] was introduced to implement the enhancement of the effective signal to highlight the amplitude variation of the SKNA signal. The TKE operator was initially proposed to compute the energy of sound [26] or to detect the onset of sound in the field of non-linear speech signal processing. It can characterize the variation degree of signal in amplitude and frequency domain as shown in Figure 3. Therefore, the amplitude-frequency variation of SKNA can be characterized by preprocessing the signal with the TKE operator.

For a given signal sequence *f*(*n*), the TKE operator can be written as:

(3) $$\varphi \left(n\right)=f^{2}\left(n\right)-f\left(n+1\right)f\left(n-1\right)$$

where *f*(*n*) is the filtered SKNA signal and *φ*(*n*) is a new discrete sequence after processing with the TKE operator, as shown in Figure 3b,c. The baseline noise is effectively suppressed. The difference between the baseline and the burst is clearer, which lays a good foundation for the later burst detection algorithm.

#### 2.3.2. Signal Segmentation

The discrete sequence was obtained through the processing of the TKE operator. First, full-wave rectification was applied to the discrete sequence. The time window method was used to obtain the envelope to shield the small fluctuations in the discrete sequence and obtain the overall trend of the signal.

The time window length was set to be n, and the envelope at the time point i was defined as the maximum sequence amplitude in the time window. Since the minimum duration of the nerve action potential is 2 ms, the default value of *n* is 2 ms if the expert does not have an extra set.

(4) $$Sknae\left(i\right)=\max\left(\varphi \left(j\right)\right),j\in \left[i-\frac{n}{2},i+\frac{n}{2}\right]$$

The characteristic of burst start/end is that the signal changes from stationary white noise signal to maximum peak value. Therefore, the start/end position of the burst can be preliminarily detected in the light of the magnitude *Sknae*(*i*) of the envelope and the derivative *d*(*i*) of the point, if:

(5) d(i)=Sknae(i)−sknae(i−1)

(6) Sknae(i)≥t and d(i)>0

(7) $$eth=\lambda \times Skna_{max}$$

where eth is the envelope threshold, which is defined as an empirical value based on the maximum value of the baseline. *λ* is an empirical parameter, which is adjusted according to different subjects. Commonly, *λ* is set to 1. That is, the maximum value of the baseline is used as the threshold of the envelope by default. Similarly, the end of the burst is defined as the envelope value less than t, and the derivative is less than 0.

Through the above operation, the start/end position of the burst was detected. However, the signal rose unsteadily, so the start/end position needs to be adjusted slightly. The significance of using the two methods is that the envelope can be used for rough segmentation. The integral signal is used to adjust the starting point position in more detail to segment the signal more accurately. The integral of *φ*(*n*) is the integral area of the sequence in unit time. It can reflect the detailed changes of sequence better than the envelope. Therefore, the interference of the signal jitter on the envelope start/end position can be reduced to obtain more accurate burst detection results. The *φ_Integral_* (*n*) is defined as:

(8) $$\varphi_{Integral\;}\left(n\right)=\frac{1}{∆t}\int_{i-\frac{1}{2∆t}}^{i+\frac{1}{2∆t}}\varphi \left(j\right)$$

where △*t* is the length of the time window. In the above process, a larger time window of signal envelope can better reflect the overall trend of the sequence. In this operation, selecting the time window △*t* as half of the envelope window n can obtain more accurate segmented positions based on rough segmentation.

The rough segmentation envelope length *L* can be calculated according to the following formula:

(9) L=EP−SP

where *SP* is the starting position of the burst and *EP* is the ending position of the burst. The range of the moving time window is one-tenth of the envelope length *L* in the adjustment step. The segmented burst area in this step is shown in Figure 3d. Therefore, the new starting and ending position is:

(10) $$SP_{new}=\min\left(\varphi_{\mathrm{Integral}\;}\left(i\right)\right),\;i\in \left[SP-\frac{1}{10}L,SP+\frac{1}{10}L\right]$$

(11) $$EP_{new}=\min\left(\varphi_{\mathrm{Integral}\;}\left(i\right)\right),\;i\in \left[EP-\frac{1}{10}L,EP+\frac{1}{10}L\right]$$

#### 2.3.3. Discrimination of Artifact Bursts

Burst in the signal was detected through the above process, including true nerve burst and false burst. Since the characteristics of the false burst are similar to the trust burst, they are difficult to distinguish based on the envelope.

SKNA signal was obtained from the body surface by standard lead. Therefore, the ECG artifact was the major noise source in SKNA. Although most energies of ECG were filtered out after a 500–1000 Hz band-pass filter, some residual energy still existed as background noise, especially in QRS complexes. Firstly, the R-peaks detection was performed on ECG using an open-source QRS detector [27]. Then, adaptive thresholds were applied to the length signal to determine the onset and duration of the QRS complexes [28]. In this way, we obtained two pieces of information: the position of the ECG artifact and the maximum width of each ECG artifact [29]. If:

(12) $$SP<Index_{R}<EP\;\mathrm{and}\;W_{qrs}<L$$

where *Index_R_* is the index of the position of the *R* wave and *W_qrs_* is the duration of the QRS complexes. That is, in case the burst area contains the position of the *R* wave and the width of the burst area is smaller than the width of the QRS complex, the burst area is supposed to be a false positive.

As shown in Figure 3, false positives resulting from short bursts in SKNA are discriminated by applying time thresholds to the start/end positions. In other words, start/end positions that had time differences shorter than Ts seconds were removed. This discriminating operation was applied after the start/end positions had been detected. This operation allowed the algorithm to have a sensitive threshold for the on-time [30]. Since the minimum duration of the nerve action potential is 2 ms [31], the default value of Ts is 2 ms if the expert does not have an extra set.

### 2.4. SKNA Energy Ratio

In [32,33], aSKNA was used to assess SKNA over a period. The length of the period depends on the length of time required for clinical analysis, 30 s [11], 5 min [12], and half an hour or more [22]. The feature can be calculated in two steps. First, iSKNA is obtained by calculating the sum of the areas under the SKNA curve in unit time, as shown in Formula (7). The unit time was set to 0.1 s in the previous research [12]. Then, the amount of SKNA can be quantified by calculating the average of iSKNA, which is defined as aSKNA.

(13) $$aSKNA=\frac{\sum^{\;}iSKNA\left(i\right)}{N}$$

where *N* is the ratio of the time window of the calculated feature to that of *iSKNA*. For example, *N* is 3000 if the time window of *aSKNA* is 5 min. Noise has a great impact on this feature, especially the baseline noise and impulse noise. Moreover, due to the differences in sensors and subjects, this feature is unstable in individual comparison. Therefore, according to the concept of signal-to-noise ratio (*SNR*), we defined a feature to estimate SNA by calculating the ratio of the detected burst sequence energy to the baseline energy. The *SKNA* energy ratio (*SKNAER*) is defined as:

(14) $$SKNAER=10\log_{10}\frac{P_{burst}}{P_{baseline}}$$

where *P_burst_* is the total energy of the nerve burst area detected after step 4 as show in Figure 3. *P_baseline_* is the total energy of the baseline area detected after step 3. It is worth noting that the discriminated artifact burst in step 4 is neither baseline nor nerve burst. It is recognized as noise and is not used to calculate the feature. To avoid error calculation caused by extreme conditions, the signal segment is discriminated as abnormal for secondary processing when it is determined to be all baseline or burst.

### 2.5. Evaluation Methods

To verify the effectiveness and accuracy of the algorithm, the data were manually labeled by an expert. The expert scrolled through the data using a custom graphical user interface tool and manually placed onsets and offsets of SKNA burst. For the annotation of burst, firstly, we recorded the start/end time of the activation action in the standard signal recording experiment. Based on the start/end time of the activation action and the relative amplitude of the signal, experts trimmed the start/end position of the burst.

The first problem is as follows: what was the detection quality of the proposed algorithm compared with the expert? This was answered with the data of Experiment 1.

To answer the first question, we obtained detection rate and precision. These measures were defined in terms of the following quantities and expressed as percentages. The detection rate was obtained to quantify the difference in the number of detected pairs of onsets/offsets and the number of pairs manually labeled by the expert. It was defined as the percentage of the number of true onset/offset pairs detected by the algorithm to the number of bursts labeled by the expert.

True positives (TPs): numbers of burst areas classified as the burst by both the expert and the algorithm.

False positives (FPs): numbers of burst areas classified as the burst by the algorithm and not by the expert.

False negatives (FNs): numbers of burst areas not classified as the burst by the algorithm and classified as the burst by the expert.

The detection rate and precision were defined as follows.

(15) $$DR=\frac{TP}{TP+FN}\times 100\%$$

(16) $$P_{+}=\frac{TP}{TP+FP}\times 100\%$$

In addition, we also calculated the coincidence to answer the first question. The coincidence was defined to compare the coincidence degree of the automatic/manual segmentation results by algorithm/expert. The coincidence was computed as the ratio of the manual segmentation length of the overlapping part of automatic segmentation length and manual segmentation length.

(17) $$CO=\frac{\min\left(EP_{a},EP_{e}\right)-\max\left(SP_{a},SP_{e}\right)}{EP_{e}-SP_{e}}$$

where *SP_a_* and *EP_a_* are the automatic segmentation of starting and ending position by algorithm; *SP_e_* and *EP_e_* are the manual segmentation of starting and ending position by expert.

### 2.6. Reference Features

The second question is what is the clinical effect of the proposed feature, and whether it is more effective in evaluating SNA than other features? The second question can be answered by using the data from Experiment 1 and 2. In Experiment 1, the CPT and the VM are the standard procedures for triggering sympathetic discharges [32,33], which can increase blood pressure, heart rate, and SNA level. In Experiment 2, hemodialysis is to purify the blood by dispersing and circulating all kinds of harmful and redundant metabolic wastes and excess electrolytes out of the body to achieve the purpose of correcting water-electrolyte and acid-base balance. As a result, pressure on the autonomic nervous system decreases, and SNA changes with the release of toxic substances. In this study, we used two methods to process data to verify the effectiveness of SKNA-based evaluation. One method was to use the proposed method to calculate the SKNAER in different periods. The other was to calculate the aSKNA of different periods without additional processing.

In order to further compare the differences in features, we calculated low frequency/high frequency (LF/HF), the standard deviation of NN intervals (SDNN), and very low frequency (vLF) based on HRV. These indicators can reflect the characteristics of SNA. The increase of LF/HF and vLF represented the increase in SNA [34]. The decrease in SDNN indicated an increase in SNA [35]. In addition, the paired t-test was performed to evaluate the difference in features before and after time.

## 3. Results

Table 2 shows the statistical results of Experiment 1 in detail. The observed burst number of the signal collected from the arm was less than that from the chest, especially in the biceps position. In addition, the false positives and false negatives of the signal from the arm were also more than those of the signal from the chest. This may be due to more EMG interference on the arm. From this point of view, the signals obtained from the chest position and the forearm may have better signal quality than that of the biceps position. The proposed algorithm was verified on standard datasets. We calculated the burst detection accuracy of signals from three acquisition positions, respectively. The results showed that the detection rate and precision of the proposed algorithm on the acquired signal from the chest were 100.0 ± 0% and 94.2 ± 5.0%, respectively. Although the detection rate and precision of the algorithm decreased on the acquired signal from the biceps and forearm, the detection rate and precision were still acceptable, 96.4 ± 5.5% and 87.3 ± 7.4%. In addition, the coincidence area was also calculated in the experiment. The coincidence of the signal from the chest was 96.4 ± 1.2%, and it was higher than that of the signal from the arm. Experimental results showed that the proposed algorithm had a satisfactory performance on the acquired signal from a different position.

Compared to the proposed method, there were more false positives and false negatives using the method with iSKNA. On the one hand, the increase of false negatives in the biceps position was more than that in other positions. In the acquisition position with high signal quality, the detection rate of the method with iSKNA was not much different from that of the proposed method. However, the detection rate of the signal collected at the biceps position was reduced to 87.1 ± 11.0%. On the other hand, the difference between the method with iSKNA and the proposed method was mainly reflected in the number of false positives, resulting in the difference in precision. Since there was no additional processing for ECG artifacts, there were a number of false positives using the method with iSKNA. The precision and CO of the method with iSKNA were much lower than those of the proposed method. In general, the proposed method had better performance in burst detection than the method with iSKNA, especially in the signal with low signal quality.

**Figure 4:**
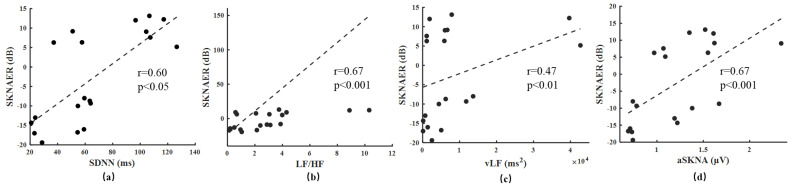
(**a**–**d**) The correlation between SKNAER and HRV features of the ten patients before and after sympathetic activation in Experiment 1.

**Figure 5:**
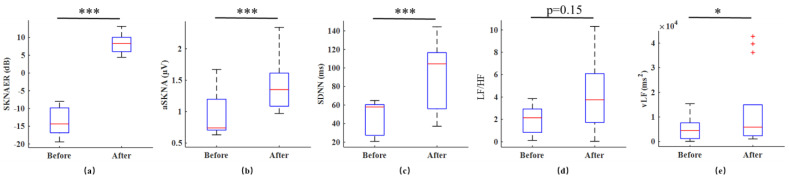
(**a**–**e**) The statistical results of the features related to the sympathetic nervous activity before and after sympathetic activation in Experiment 1. * *p* < 0.05, *** *p* < 0.001.

Figure 4a–d indicate the correlation between SKNAER and the other features of the ten patients before and after sympathetic activation in Experiment 1. The results showed that the SKNAER was positively correlated with SDNN (r = 0.60), LF/HF ratio (r = 0.67), vLF power (r = 0.47), and aSKNA (r = 0.67). Figure 5a–e show the box diagram of the features related to the SNA before and after sympathetic activation in Experiment 1. The HRV features and SKNA features of sympathetic activation one minute before and one minute after were calculated. All features showed an upward trend after sympathetic activation. For the HRV features, SDNN increased from 48.75 to 93.19 ms (*p* < 0.001). From the perspective of this feature, SNA showed a downward trend. vLF increased from 5510.96 to 12,213.52 ms^2^ (*p* < 0.05), and three outliers occurred in the experiment. LF/HF increased from 1.94 to 4.27 (*p* = 0.15). For the SKNA features, aSKNA increased from 0.91 to 1.42 µV (*p* < 0.001) and SKNAER increased from −13.76 to 8.29 dB (*p* < 0.001). The calculated values of SKNAER before and after sympathetic activation had no overlap. That is, they were almost unaffected by individuals.

**Figure 6:**
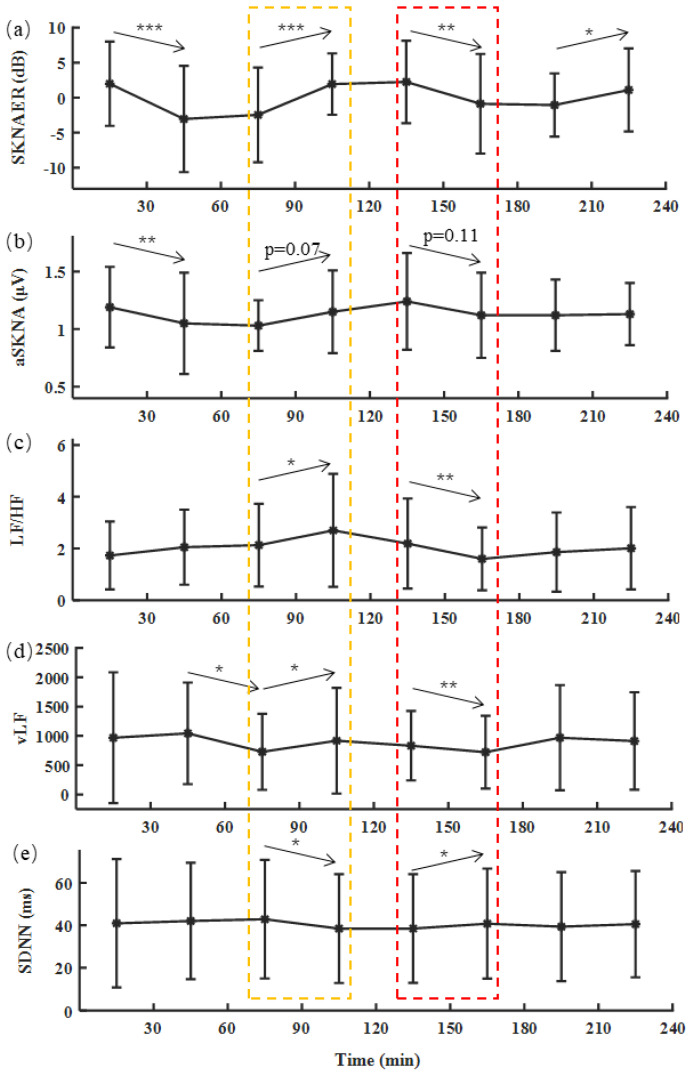
(**a**–**e**) The trend of the SKNA and HRV features in the twenty patients during four-hour hemodialysis. * *p* < 0.05, ** *p* < 0.01, *** *p* < 0.001.

The whole data process of renal dialysis for each patient lasted about 4 h. Taking 30 min as a window, data were divided into eight periods. Figure 6 shows the trend of SKNA and HRV features during dialysis in 20 patients with renal failure in the clinical experiment. The SKNA and HRV features of each period were calculated to assess the SNA of renal dialysis patients. For the SKNA features, these two features decreased the second time, aSKNA from 1.19 to 1.05 µV (*p* < 0.01), SKNAER from 1.99 to −3.04 dB (*p* < 0.001). Then, these features increased in the fourth time period, aSKNA from 1.03 to 1.15 µV (*p* = 0.07), SKNAER from −2.45 to 1.94 dB (*p* < 0.001), and began to decline in the sixth time period, aSKNA from 1.24 to 1.12 µV (*p* = 0.11), SKNAER from 2.25 to −0.87 dB (*p* < 0.01), and remained stable until the end of the operation.

For the HRV features, LF/HF maintained an upward trend in the first two hours, especially the fourth period, from 2.13 to 2.70 (*p* < 0.05), and dropped from 2.19 to 1.60 in the sixth period (*p* < 0.01), and then finally increased, reaching 2.01 in the eighth period. vLF fluctuated greatly in the first two hours. It decreased from 1043.50 to 728.80 in the third period (*p* < 0.05) and quickly increased to 918.3 in the fourth period (*p* < 0.05). The trend in the last two hours was similar to that of LF/HF, which decreased first and then increased. SDNN rose continuously in the first three periods and fell from 42.88 to 38.47 ms in the fourth period. It increased in the sixth period, from 38.49 to 40.79 ms, and remained stable. The common trend of the HRV features was that there was a significant difference in the fourth period, indicating the activation of SNA. There was also a significant difference in the sixth period, indicating inhibition of SNA. The variation tendencies of HRV features were consistent with those of aSKNA and SKNAER. However, SKNAER had a more significant difference than aSKNA in evaluating SNA at different times, especially in the fourth and sixth periods.

## 4. Discussion

In this work, we developed a burst area detection algorithm and verified the accuracy of the standard sympathetic nerve activation experiments, including the Valsalva experiment and the CPT experiment. We obtained SKNA signals by placing three groups of electrode sensors on the human surface. The data were automatically detected by the proposed algorithm and manually labeled by experts receptively. Experimental results indicated that the consistency of the detected burst area between the algorithm and the expert was high. However, the number of burst areas observed from different positions was different, and the burst number from the biceps was smaller than that of the chest. In other words, the proposed algorithm can effectively locate the burst area, but it cannot further decouple the mixed noise and neural signals. Although the SKNA signal originates from ganglion, the effective information obtained by the signal was different due to the influence of the interference. Preprocessing multichannel signals with principal component analysis and other methods may lay a better foundation before burst segmentation.

The accuracy of burst segmentation was verified on the sympathetic activation dataset. The results showed that the detection rate on the signal of the chest reached 100%. Although the detection rate decreased on the signal of the forearm and biceps, it was also greater than 95%. Therefore, the proposed algorithm showed good performance in different acquisition positions. In addition, the coincidence degree greater than 95% indicated that the sensitivity to detect the burst area was close to the manual label of the expert. Compared with the method with iSKNA, the advantages of the proposed method were the improvement of detection rate on low signal-to-noise ratio signals and the discrimination of false positives. However, due to the complex neural changes and other confounding factors in the experiment, the number of false positives was not satisfactory enough, especially for the acquisition signal at the biceps. This may be affected by the accuracy of the QRS complexes’ detected algorithm and EMG artifact, which led to the unrecognized false burst. Further analysis of the difference between the true and false burst may provide a new train of thought to reduce the misjudgment rate.

SKNA-based SNA evaluation has great potential in pathogenesis research such as atrial fibrillation [32] and myocardial infarction [22]. The SNA evaluation is often quantified by the estimation of mean burst amplitude [36], total burst amplitude [37], or burst area [38]. However, direct calculation of the mean burst amplitude, such as aSKNA [22] [32], greatly impacted the evaluation of SNA because of the signal noise. The outlier of aSKNA further proved this point during hemodialysis. We discriminated and removed the non-typical burst after detecting the burst area and calculated the burst area energy ratio to assess the SNA. This feature had better significance in reflecting the trend of the SNA during the Hemodialysis experiment. Observational results of the hemodialysis experiment showed that the SNA gradually decreased in the second period, which was the patients’ process from dynamic to static. It increased in the fourth period. This is the period when the most malignant clinical events may occur. In the sixth period, the sympathetic nerves returned to calm. The patients gradually completed dialysis for harmful substances in the body during this period. In the eighth period, the SNA increased, and the patients’ activity increased at the last moment. From the perspective of aSKNA, except for the second period, the sympathetic nerve of the patient did not change significantly compared with the previous time. However, we observed more time-to-time differences from SKNAER. From this point of view, it can help us better understand the changes in the sympathetic nerves of patients during hemodialysis.

For Experiment 1, VM and CPT are standard sympathetic stimulation procedures. Results showed that there was no significant difference between HRV-based features and SKNA-based features in indicating SNA. It is worth noting that although SDNN showed significant changes before and after sympathetic stimulation procedures, the indicated SNA was actually the opposite. Although SDNN can effectively indicate the changes in the sympathetic nerve in most clinical studies, it is difficult to explain in some specific clinical scenes [39], especially in short-term measurement. It may be because there are many interference factors for RR interval in short-term analysis, such as false heartbeat [40] and algorithm error. Therefore, SKNA-based features were better than HRV-based features in the short-term analysis of SNA. Furthermore, the values of SKNAER before and after sympathetic activation did not overlap, while those of other features overlapped to a certain extent. In other words, SKNAER can better avoid individual differences compared to the other features. This can help us compare the SNA of different individuals, not just individuals themselves. According to the experimental results of this study, the frequency domain index of HRV may have a better correlation and interpretation with SKNAER in short-term analysis, such as LF/HF.

For Experiment 2, the trends of all features were not completely consistent in reflecting changes in SNA. However, these features showed the same change trend in some specific time periods, the fourth and sixth time periods, respectively. In other words, although the two kinds of features were calculated based on different signals, they were consistent in reflecting the trend of SNA at some levels. For the SNA evaluation, many features provided indirect explanations. However, these features sometimes did not always show the same trend [41,42]. This may be because the signal was doped with various interference factors, resulting in inaccurate interpretation. For example, HRV features were calculated based on RR intervals. The RR interval is often affected by the accuracy of the R-wave-detecting algorithm. In addition, patients with atrial fibrillation and premature beats also have a negative impact on the calculation results because of their special heart rhythm. Similarly, SKNAER is also affected by these factors. The wrong R-wave location result may lead to the misjudgment of the SKNA burst, which reduces the precision of the algorithm, especially in the signal with low signal quality. However, the positive outcome is that we discriminated and removed some interference in the calculation to provide a more accurate evaluation of SNA. In addition, SKNAER is calculated based on the energy of burst and baseline, so the influence of R-wave positioning accuracy on SKNA is indirect and less than that of the HRV index. With the advanced QRS positioning algorithm, the influence of R-wave positioning error on the SKNA feature can be further reduced. Furthermore, we established an evaluation method based on SKNA. Since SKNA is the real-time signal transmitted from the sympathetic nerve to the body surface, while ECG is the change of potential cardiac signal caused by the sympathetic nerve affecting cardiac function, thus the method based on SKNA is more direct than that of ECG in evaluating SNA. This also provides an auxiliary basis for the analysis of disease mechanisms.

The findings from this study should be considered in light of several factors. First, the proposed feature assumes that there is sympathetic activation in the measured data segment. The feature may output an error in case there is no burst area in the data segment or the noise covers the burst. Second, the shape and frequency of the burst were not considered for the proposed feature. This more in-depth information should be further processed and characterized to be applied to some specific clinical scenarios. Last, although the TKE factor is used to enhance the amplitude-frequency change of the signal, this operation may also enhance the amplitude-frequency change of some artifacts, resulting in false positives. Research on the conduction mode of nerve signals to the body surface and then distinguishing between effective nerve burst and noise is conducive to further finding effective information from SKNA. In addition, SKNA is greatly affected by motion artifacts The impact comes from the electrodes used to collect signals and the lack of appropriate standards to distinguish effective signals from motion artifacts. This may limit the further application of SKNA. Therefore, SKNA can be used as an effective supplement rather than a substitute for HRV in some specific scenarios, especially in short-term measurement.

## 5. Conclusions

This paper proposed an SNA evaluation method based on SKNA burst area detection. This method exhibited good performance in terms of detection rate, concordance, and precision on the sympathetic activation dataset manually labeled by the experts. The trend of SNA during hemodialysis was analyzed quantitatively based on the detected burst area. The results showed that SKNAER has a consistent trend in evaluating SNA compared with HRV features. Moreover, it had a more significant difference in the long-term SNA evaluation than aSKNA, consistent with the HRV features. The automatic burst detection algorithm proposed in this work can accurately locate the position of the active SNA, which is helpful to evaluate SNA more accurately. With further development, this new modality could play an important role in continuous monitoring of the autonomic nervous system status, as well as preventing correlated diseases. Future work will explore more useful physiological features for evaluating the autonomic nervous system, such as time-frequency analysis and non-linear dynamic evaluation.

## Figures and Tables

**Table 1:** Summary of demographic information of subjects that participated in experiments.

	Experiment 1		Experiment 2	
	Mean	Standard Deviation	Mean	Standard Deviation
Age/years	25.1	4.6	58.9	14.6
Height/cm	173.2	6.5	170.2	10.3
Weight/kg	71.0	13.6	70.5	13.9
Weight/kg (after dialysis)			67.9	13.6
Cohort size	10		20	

**Table 2:** The mean, standard deviation, and confidence interval (90% CI) of detection rate, coincidence, and precision of the proposed algorithm on the acquired signal of Experiment 1. * *p* < 0.05, ** *p* < 0.01, *** *p* < 0.001.

Position		TP	FN	FP	DR (%)	*p*-Value	CO (%)	*p*-Value	P+ (%)	*p*-Value
Ch1 Chest	Proposed method	159	0	9	100.0 ± 0 [100.0 100.0]	0.18	96.4 ± 1.2 [95.6 97.2]	***	94.2 ± 5.0 [91.9 97.9]	***
	With iSKNA [12]	157	2	103	98.8 ± 2.6 [97.2 100.2]		92.2 ± 1.7 [91.4 92.8]		59.9 ± 3.6 [58.2 62.4]	
Ch2 Biceps	Proposed method	111	3	18	96.4 ± 5.5 [95.6 99.3]	*	92.3 ± 2.1 [91.6 92.9]	***	87.3 ± 7.4 [85.3 89.4]	***
	With iSKNA [12]	100	14	47	87.1 ± 11.0 [84.5 92.5]		87.6 ± 2.4 [87.0 88.6]		67.6 ± 9.3 [63.9 70.5]	
Ch3 Forearm	Proposed method	147	2	10	98.7 ± 3.2 [97.0 100.3]	0.34	94.2 ± 1.3 [93.3 94.9]	***	93.7 ± 2.6 [92.3 95.4]	***
	With iSKNA [12]	146	3	94	97.8 ± 5.8 [94.2 101.0]		91.0 ± 0.9 [90.5 91.5]		60.5 ± 5.8 [57.1 63.8]	
Summary	Proposed method	417	4	46	98.2 ± 3.9 [97.7 99.6]	**	94.3 ± 2.3 [93.6 95.0]	***	91.8 ± 6.2 [89.9 93.9]	***
	With iSKNA [12]	403	18	244	94.5 ± 8.9 [91.7 97.2]		90.3 ± 2.6 [89.5 91.0]		62.8 ± 7.3 [60.4 65.0]	

## Data Availability

Not applicable.
